# Amivantamab Plus Lazertinib and Platin-Based Chemotherapy Plus Osimertinib in EGFR-Mutant NSCLC: How to Choose Among Them and When Is Monotherapy with Osimertinib Still the Best Option?

**DOI:** 10.3390/curroncol33010054

**Published:** 2026-01-17

**Authors:** Paolo Maione, Francesco Jacopo Romano, Cesare Gridelli

**Affiliations:** 1 Division of Medical Oncology, S.G. Moscati Hospital, 83100 Avellino, Italy; 2 Division of Medical Oncology, Cardarelli Hospital, 80131 Naples, Italy

**Keywords:** EGFR-mutant NSCLC, Osimertinib, Amivantamab, Lazertinib, platin-based chemotherapy, shared decision making

## Abstract

In the last year, great advances in the treatment outcomes of advanced EGFR-mutant NSCLC have been achieved. Two combination regimens, amivantamab plus lazertinib and platin-based chemotherapy plus osimertinib, have yielded, in the phase III randomized trials named MARIPOSA and FLAURA 2, statistically and clinically significant improvements in overall survival compared with monotherapy with osimertinib. However, translation to clinical practice of these relevant results is challenging for two main reasons. The first is that we have no evidence-based tools to choose among the two combinations, except their different safety profiles. The second is that combinations are significantly more toxic than osimertinib alone. Thus, osimertinib remains an effective treatment with an excellent safety profile, perhaps to be considered as still the best option in the majority of elderly patients and in all patients that do not intend to trade-off an excess of toxicity with survival prolongment. The safety and efficacy characteristics of the three treatment options are the basis for a patient-tailored treatment choice, but in a significant proportion of patients, a personal and intimate approach to quality of life and survival prolongment is to be considered the main driver within a well-structured shared decision-making process.

## Simple Summary

Lung cancers harbouring epidermal growth factor receptor mutations are characterized by treatment options that are better in terms of efficacy and quality of life compared with the majority of different types of lung cancers. Treatment has been based on oral drugs for many years, the last generation of them named osimertinib and lazertinib. In the last two years, new combination (two- or three-drug) treatments have been developed which are more effective, but also more toxic, and based on intravenous or subcutaneous drugs to be administered in hospital and not at home. Thus, the current challenge is the selection of the right treatment for each individual patient, based on clinical characteristics but also on the preference of the patients. Patients, sharing the decision with their oncologists, can choose more effective and more toxic combination treatments or traditional oral single-agent treatments with a milder toxicity profile and often better quality of life.

## 1. Introduction

Activating mutations in the epidermal growth factor receptor (EGFR) gene is a relatively frequent event in patients with advanced non-squamous non-small-cell lung cancer (NSCLC), occurring in 15 to 50% of cases. This molecular event is associated with particular clinical characteristics, including very good treatment outcomes with EGFR tyrosine kinase inhibitors (TKIs) [1,2]. The most clinically influential and also more frequent EGFR mutations are exon 19 deletions (Ex19del) and exon 21 codon p.Leu858Arg (L858R) substitutions, which together account for 85 to 90% of all EGFR mutations [3]. In the last two years, exciting advances have been made in the treatment of epidermal growth factor receptor (EGFR)-mutant advanced non-small-cell lung cancer (NSCLC). EGFR-mutant NSCLC may be considered the queen of oncogene-addicted NSCLC diseases, because it was the first to be discovered [4]. Moreover, it has been treated in the first-line setting with an oral monotherapy based on EGFR tyrosine kinase inhibitors (TKIs) for about twenty years, with excellent efficacy and safety profile outcomes [5,6,7], but now the door is open for more efficacious combination treatments. The MARIPOSA and FLAURA 2 phase III randomized trials have demonstrated that the combinations of amivantamab (an EGFR-MET-bispecific antibody) plus lazertinib (an oral third-generation EGFR-TKI) and of platin-based chemotherapy plus osimertinib (an oral third-generation EGFR-TKI) are superior in terms of progression-free survival (PFS) and overall survival (OS) compared to the previous standard of care, osimertinib alone [8,9,10,11]. This history would be simple and univocally positive, especially with the establishment of two new, better standards of care, but for the first time in the treatment of oncogene-addicted NSCLC, these results have left the majority of oncologists confused and doubtful. In fact, both of the above-mentioned trials demonstrated not only better efficacy outcomes but also worse safety profiles for the new combination treatments, including with parenteral administration, in a clinical context where the oral route of administration and an excellent quality of life have characterized the standard approach for the last twenty years. Thus, the new challenge for this clinical context is when to choose a combination therapy over monotherapy with osimertinib, and in cases of choosing a combination, which one to select among the two proposed in the MARIPOSA and FLAURA 2 trials. The aim of this paper is to contribute to the challenging process of clinical decision making in the first-line treatment of advanced EGFR-mutant NSCLC by describing the three main treatment options and also by proposing all the points of view from which the scenario can be looked at, going beyond the mere results of clinical trials. Rarely in cancer treatment, and specifically never in the treatment of lung cancer, has it happened that such impressive improvements in efficacy outcomes (about a ten-month advantage in median survival [11]) have been perceived with such diffidence and criticism. The reason for this reaction is that innovations have arrived in a clinical context characterized by a progressive improvement not only in survival outcomes but also in safety profile and quality of life from chemotherapy to third-generation EGFR-TKIs [5,7]. Thus, accepting a step back in toxicity profile is now quite hard for physicians. The importance of enhancing this type of discussion stands in the need to prepare oncologists to make the right decision for each individual patient, always with their ears open to patients’ voices for real shared decision making.

## 2. Osimertinib as Monotherapy

The approach of administering a single oral drug has produced excellent outcomes with first- and second-generation EGFR TKIs. Gefitinib, erlotinib, and afatinib have been proven to improve progression-free survival (PFS) in patients with advanced EGFR-mutant NSCLC compared with platinum-based chemotherapy [5,6,12]. This evidence has been further confirmed in a meta-analysis of phase III randomized trials on the comparison between first-generation EGFR TKIs (gefitinib and erlotinib) and chemotherapy as first-line treatment [13]. The history of single-agent treatment of EGFR-mutant NSCLC was further changed and improved with the clinical development of the third-generation EGFR–TKI osimertinib [14,15]. Osimertinib is an oral drug, like its predecessors, but it has the peculiar characteristics of being both an irreversible and selective inhibitor of EGFR-TKI. Osimertinib, a third-generation EGFR-TKI, was demonstrated to be superior as a first-line treatment of advanced EGFR-mutant NSCLC in terms of efficacy and safety profile as compared to first-generation EGFR-TKIs in a phase III randomized trial named Flaura [7]. Osimertinib was administered to 556 patients, while 279 patients were treated with erlotinib and 277 with gefitinib. Osimertinib performed better than erlotinib and gefitinib in terms of median progression-free survival (18.9 months versus 10.2 months, respectively), in terms of response rate (80% versus 76%, respectively) and disease control rate (97% versus 92%, respectively). Moreover, patients treated with osimertinib experienced a clinically and statistically longer median survival time (38.6 months for patients treated with osimertinib versus 31.8 months for those treated with erlotinib or gefitinib; hazard ratio for death, 0.80; *p* = 0.046). Subgroup analysis of overall survival revealed that the advantage given by treatment with osimertinib was reported for the whole trial population. Elderly patients were included in the trial without upper age limits, with a median age of 64 years and a surprising age range of 26–93 years. Overall survival advantage was reported both for patients aged less and more than 65 years, but the HR was statistically more robust for patients aged 65 years or less. As mentioned before, the superiority of osimertinib over previous-generation EGFR-TKIs was also due to the milder toxicity profile of osimertinib versus erlotinib and gefitinib, especially in terms of skin toxicity (rash or acne, 58% versus 78%). Specific data in the elderly subpopulation were not reported. Thus, the universe of single-agent treatment of advanced EGFR-mutant NSCLC with EGFR-TKIs, already to be considered successful compared with the outcomes of non-oncogene-addicted NSCLC, had achieved the utmost level in terms of efficacy and safety profile with the third-generation EGFR TKI osimertinib. However, from the patient point of view, this is not enough, because resistance to EGFR TKIs always occurs, followed by progressive disease and death.

## 3. Amivantamab Plus Lazertinib

Amivantamab is an EGFR-MET-bispecific antibody that not only inhibits EGFR and MET pathways but also stimulates immunologic reaction against tumour cells harbouring EGFR-activating mutations [16,17]. Due to the binding of this antibody to the extracellular domain of EGFR, this innovative drug is capable of being independent of all resistance mutations developing in the intracellular domain of the receptor during cancer progression. Obviously, its activity against MET pathway hyperactivation also prevents and/or delays one of the most frequent and influent mechanism of resistance to EGFR-TKIs [18]. Amivantamab, as a first-line treatment of advanced EGFR-mutant NSCLC, has been developed in combination with lazertinib, an oral third-generation EGFR-TKI like osimertinib [19]. Amivantamab and lazertinib act synergistically against EGFR pathologic activity, producing therapeutic effects in patients whose tumours harbour EGFR-activating mutations [20]. Thus, the phase III randomized trial named MARIPOSA [8] was developed to test the superiority of the combination of amivantamab plus lazertinib versus osimertinib alone, with the hope that the combination would proactively counteract mechanisms of resistance to osimertinib, positively affecting the survival of patients with advanced EGFR-mutant NSCLC. In the MARIPOSA trial, the combination of amivantamab plus lazertinib was demonstrated to achieve a significantly longer median PFS compared with osimertinib alone (primary endpoint of MARIPOSA trial; 23.7 versus 16.6 months, respectively, with an HR of 0.70 and *p* < 0.001). More importantly, in the MARIPOSA trial the combination of amivantamab and lazertinib was also demonstrated to be superior compared to osimertinib alone in terms of overall survival [9]. At a median follow up of 37.8 months, the resulting median survival was 36.7 months for osimertinib and was not reached for the combination, with a hazard ratio for death of 0.75 (95% CI, 0.61–0.92; *p* < 0.005). Moreover, the survival rate at three years was 60% in the amivantamab plus lazertinib group, compared with 51% in the osimertinib arm. In the MARIPOSA trial, patients were included without an upper age limit, with a median age of 64 years and an age range of 25–88 years in the amivantamab–lazertinib arm. The overall survival data for predefined subgroups revealed a better HR of 0.53 in favour of the combination therapy in patients aged less than 65 compared to those > 65 years. However, an impressive HR of 0.75 favouring the combination was reported also for patients aged less than 75 years. However, the combination of amivantamab and lazertinib was proven to be more toxic as well as more effective in terms of PFS and OS. Toxicity data specific for the elderly subpopulation included in the trial were not reported. As previously reported with double EGFR blockade with an antibody and a TKI [21], the combination was found to be more toxic in terms of rate of grade 3 or higher adverse events, primarily paronychia and skin rash (75% versus 43% for amivantamab plus lazertinib versus osimertinib alone, respectively). This appears to be an obvious consequence of the inhibition of the EGFR pathway on both sides of the cytoplasmic membrane. Infusion-related reactions and venous thromboembolic adverse events occurred in 63% and 37% of the patients treated in the MARIPOSA trial with amivantamab–lazertinib and osimertinib, respectively. The observation that the great majority of infusion reactions and of thromboembolic events occurred in MARIPOSA trial at the beginning of treatment with amivantamab plus lazertinib, specifically during the first 4 months, suggests that prophylaxis with steroids and anticoagulants may significantly reduce the clinical presence of this safety issue. In fact, a phase II trial named SKIPirr was performed to clarify this relevant issue and showed that dexamethasone 8 mg administered orally bis in die in the two days preceding cycle 1 day 1 and 1 h before administering amivantamab yielded an about 3-fold reduction in infusion-related reaction rate compared to the standard clinical approach (from 67.4% to 22.5%) [22]. In the SKIPirr trial, elderly patients were also enrolled (median age 63 years with a range of 32–82 years), but specific data for this subpopulation were not reported. In order to prevent dermatologic adverse events during amivantamab treatment, the phase 2 COCOON study evaluated early dermatologic management in patients treated with first-line amivantamab and lazertinib [23]. The COCOON study randomized participants with previously untreated, EGFR-mutant, locally advanced or metastatic NSCLC undergoing intravenous amivantamab plus oral lazertinib treatment with enhanced dermatologic management (COCOON DM) and standard-of-care treatment (SoC DM) per local guidelines. COCOON DM included oral tetracycline (doxycycline or minocycline 100 mg twice daily up to 3 months), clindamycin 1% (on scalp daily; weeks 13 up to one year), chlorhexidine 4% (on fingernails and toenails daily), and a ceramide-based moisturizer (on body and face at least daily). Grade 2 or higher dermatologic adverse events occurred in 42% of patients undergoing enhanced dermatologic management versus 75% of the SoC DM group (OR, 0.24; 95% confidence interval, 0.13–0.45; *p* < 0.0001). In the COCOON trial, patients were also included without an upper age limit, with a median age of 63 years and a range of 28–83 years, very similar to the SKIPirr trial population. However, data were not presented separated for age; thus, we have no specific data for elderly patients. It is important to underline that in the PALOMA-3 phase III randomized trial, subcutaneous amivantamab plus lazertinib recently demonstrated non-inferiority to intravenous amivantamab plus lazertinib, with a significant reduction in the rate of infusion-related reactions (13% vs. 66%) compared with intravenous amivantamab [24]. As a consequence, subcutaneous amivantamab will soon become the preferred way of administering this drug due to the significant mitigation of its safety profile. In conclusion, the combination of amivantamab plus lazertinib produced significant and clinically relevant PFS and OS improvements over osimertinib alone, with an absolute 3-year benefit in the probability of being alive of 9%. This relevant efficacy outcome is at the cost of significant, symptomatic, early, and long-lasting toxicity, requiring intensive medical prevention and management and impacting the daily quality of life of patients during a period when the disease is generally well-controlled.

## 4. Platin-Based Chemotherapy Plus Osimertinib

FLAURA2 was a phase III randomized trial aimed at evaluating the addition of platinum-based chemotherapy to osimertinib [10]. It enrolled 557 patients with previously untreated, locally advanced, or metastatic NSCLC positive for activating EGFR mutations (Ex19del or L858R). Patients were required to have a WHO performance status of 0 or 1, and individuals with stable, treated brain metastases were eligible. Patients were randomized in a 1:1 ratio to osimertinib monotherapy (80 mg orally once daily) or osimertinib at the same dose plus chemotherapy consisting of pemetrexed (500 mg/m^2^) plus either cisplatin (75 mg/m^2^) or carboplatin (AUC 5) every 3 weeks for four cycles, followed by maintenance therapy with osimertinib plus pemetrexed every 3 weeks. The primary endpoint was PFS, assessed by investigators according to RECIST criteria. Key secondary endpoints included OS, objective response rate, duration of response, time to subsequent treatment, safety, and health-related quality of life. Treatment with cisplatin or carboplatin plus pemetrexed plus osimertinib was demonstrated in the FLAURA 2 trial to be superior compared to osimertinib alone in terms of median PFS (25.5 months versus 16.7 months, respectively, with an HR of 0.62, *p* < 0.001). This advantage in terms of PFS, favouring the combination, was extended to all the subgroups of patients enrolled in the FLAURA 2 trial. Specifically, concerning the type of activating mutation, the superiority in PFS of the combination appeared to be higher for patients whose tumours harboured the L858R mutation (24.7 versus 13.9 months) compared with those harbouring exon 19 deletion (27.9 months versus 19.4 months). The combination of chemotherapy plus osimertinib appeared to be superior compared to osimertinib alone to a greater degree in another subgroup of patients with poor prognosis: those with brain metastases at the beginning of the trial (median PFS results of 24.9 months for patients treated with the combination versus 13.8 months for those treated with osimertinib alone). The combination of chemotherapy plus osimertinib was also demonstrated to be superior to osimertinib alone in patients without central nervous system involvement (median PFS of 27.6 months versus 21.0 months, respectively), but the difference in results was less than that in patients with brain metastases at baseline. In the FLAURA 2 trial the combination of chemotherapy plus osimertinib was also more active than osimertinib alone (response rate of 83% versus 76%, respectively) and produced a longer median response duration (24.0 versus 15.3 months, respectively). The latest FLAURA2 trial data, presented in September 2025 at WCLC, show in the final overall survival analysis that combining osimertinib with chemotherapy significantly improves overall survival compared with osimertinib alone [11]. The combination therapy resulted in a median OS of 47.5 months compared to 37.6 months for osimertinib alone, a nearly 10-month benefit, with a 23% reduction in risk of death—HR 0.77 (95% CI, 0.61–0.96; *p* = 0.02). The 36-month survival rate was 63.1% for the combination therapy versus 50.9% for monotherapy. At four years, the survival rate was 49.1% for the combination versus 40.8% for monotherapy. In the FLAURA 2 trial, elderly patients were included without an upper age limit, with a median age in the osimertinib plus chemotherapy arm of 61 years and an age range of 26–83 years—a slightly younger population than in the MARIPOSA trial. In the FLAURA 2 trial there were no subgroup analyses reported for the different age groups like the authors performed in the MARIPOSA trial. Moreover, the toxicity profile of osimertinib plus chemotherapy was not described separately for age subgroups, thus making it impossible to draw efficacy- and toxicity-specific hypotheses for treating elderly patients with chemotherapy plus osimertinib. The improvement of efficacy outcomes was counterbalanced by a worse toxicity profile of platin plus pemetrexed plus osimertinib compared with the excellent safety profile of the single-agent treatment with osimertinib. The advantage in toxicity profile favouring osimertinib presented mainly in terms of the rate of grade 3 or higher adverse events, appearing in 27% of patients treated with osimertinib and 64% of those treated with the combination of chemotherapy plus osimertinib. The difference in toxicity profile favouring osimertinib alone was expected due to the higher rate of hematologic toxicity reported for the combination (71%) versus only 24% in patients treated with osimertinib alone. Moreover, more patients in the combination arm experienced adverse events leading to discontinuation of osimertinib (12%) compared to the monotherapy arm (7%). However, the safety profile of the combination regimen was consistent with the known toxicities of each drug, with no new safety alerts found. The occurrence of chemotherapy-induced toxicities, such as myelosuppression, nausea, and fatigue, was considered manageable within the clinical practice (Table 1).

## 5. Clinical and Molecular Factors Useful in Treatment Choice

### 5.1. Combinations Versus Osimertinib

Several clinical and molecular factors have been studied, aiming to understand if and when a de-escalation of treatment intensity is feasible for EGFR-mutant NSCLC patients [25]. However, current data show only a prognostic and not a predictive role for some of them [26]. The presence versus absence of liver and/or brain metastases, presence versus absence of TP53 co-mutation, positive versus negative circulating tumour DNA, and tumour burden seem to show subgroups of patients with the worst prognoses independently of treatment in both the MARIPOSA and FLAURA 2 trials [27,28]. In other words, the improvement of efficacy outcomes achieved with the more toxic combinations is also observed in the subgroups of patients with better prognoses, and this type of information is derived both from the MARIPOSA and FLAURA 2 trials. Thus, when communicating with our patients, subgroup analyses can be used only to better clarify prognosis. When discussing a clinical case characterized by the absence of negative prognostic factors, the only conclusion we can transfer to our patients is that osimertinib alone has a high probability of achieving long PFS and consequently a longer OS time.

### 5.2. Platin-Based Chemotherapy Plus Osimertinib Versus Amivantamab Plus Lazertinib

It is important to underline that cross-trial comparisons among the FLAURA 2 and MARIPOSA trials are indirect and only hypothesis-generating. No direct comparisons among the two combination therapies are available; thus, only indirect comparisons and discussions can be made. Concerning the presence at baseline of central nervous system (CNS) involvement, some considerations can be made. In the FLAURA 2 trial, patients with stable lesions (asymptomatic or after radiotherapy) were allowed to participate, and the benefit in PFS favouring chemotherapy plus osimertinib over osimertinib alone was also reported in these patients with CNS involvement (also with a higher magnitude of benefit, with an HR of 0.47 versus 0.75) [10,11]. The CNS activity of osimertinib is well-known, because it penetrates the blood–brain barrier, while pharmacokinetic studies have suggested only limited CNS penetration for cisplatin, carboplatin, and pemetrexed. However, the presence of CNS metastases might facilitate brain penetration of chemotherapy, owing to disruption of the blood–brain barrier, explaining this beneficial clinical effect in the FLAURA 2 trial. In the MARIPOSA trial, patients with stable brain metastases were also included, and benefits in PFS and OS favouring the combination of amivantamab plus lazertinib versus osimertinib were reported in patients both with and without brain lesions (with a better OS HR in patients with brain metastases) [8,9]. In the MARIPOSA trial the benefit in intracranial PFS favouring amivantamab–lazertinib lead to a 3-year landmark intracranial PFS of 36% versus 18%, with a better intracranial duration of response of 35.7 versus 29.6 months [8,9]. Concerning the type of EGFR mutation (Ex19del versus L858R), in subgroup analysis of the MARIPOSA trial, the combination of amivantamab plus lazertinib seems to offer a greater benefit than osimertinib in patients with Ex19del mutations [8,9]. On the contrary, in the FLAURA 2 trial, subgroup analysis for the two different types of mutations shows an equal benefit in favour of the combination of chemotherapy plus osimertinib versus osimertinib, with a superimposable HR of 0.76 in the OS outcome [10,11]. At the moment we cannot state that one combination is preferable over the other in patients with brain metastases or in those with a certain type of EGFR mutation (both perform better than osimertinib alone). It is only hypothetical that chemotherapy plus osimertinib may perform better in patients whose tumours harbour L858R mutations, while both combinations are particularly useful in patients with brain metastases (with amivantamab plus lazertinib reporting the best OS outcomes in subgroup analyses).

The similar clinical outcomes in the two studies (MARIPOSA and FLAURA 2) suggest that the choice of the best treatment may be driven more by the toxicity profile than by the actual difference in efficacy [8,9,10,11]. Skin toxicity requiring antibiotic prophylaxis and the obligation to practice anticoagulant therapy to prevent deep venous thrombosis can be obstacles in patients with multiple comorbidities. Indeed, typical patients suffering from this malignancy often require other medications, and therefore a therapeutic regimen requiring the addition of further drugs may reduce compliance with treatment. Moreover, the COCOON regimen, although proven to reduce the rate of skin toxicity, must be considered together with the patient’s comorbidities and medications. In fact, diarrhea can be triggered by the concomitant use of anti-EGFR drugs and tetracyclines because tetracyclines can alter the intestinal bacterial flora, causing the proliferation of Clostridium difficile. Nevertheless, platinum–pemetrexed plus osimertinib may not be indicated in elderly patients with poor bone marrow reserve and reduced glomerular filtration rate. Another topic to remember when choosing a first-line combination treatment is the second-line treatment that each combination allows. In terms of the sequence of treatments, we may affirm that the combination from the MARIPOSA trial is favoured. In fact, if treated with platin-based chemotherapy plus osimertinib as first-line treatment, patients will be treated in the second-line setting with docetaxel when systemic progression occurs. For patients treated in the first-line setting with amivantamab plus lazertinib, the second-line treatment options include the more complete treatment choice of platin-based chemotherapy.

### 5.3. Elderly Patients, Poor PS Patients, and Real-World Data

Although both the MARIPOSA and FLAURA 2 trials enrolled patients without an upper age limit, the proportion of those aged over 75 was 12% (n = 104/858) in the MARIPOSA trial (the same information for FLAURA 2 trial is not available but we think it should be similar due to the platin-based chemotherapy included in the trial) [8]. Thus, in our opinion, MARIPOSA and FLAURA 2 results should not be acritically generalized to the entire elderly population aged more than 75 years, but they should be applied to patients with clinical characteristics (especially in terms of PS and comorbidities) similar to those of elderly patients enrolled in these two trials [29]. The message that the combinations are superior to osimertinib alone both in young and in the majority of elderly patients should not be given, in our opinion. For EGFR-mutant NSCLC, in our opinion, a commonsense approach specific to elderly patients should be used. In other words, osimertinib is to be considered an excellent treatment for advanced NSCLC in elderly and very elderly patients (high efficacy with a safety profile particularly suitable to that age), and an attempt to increase efficacy at the cost of an increase in toxicity in such a population, very often characterized by polypharmacotherapy, frailty, and lack of social and familiar support, may result in disadvantages. In our opinion, amivantamab plus lazertinib and platin-based chemotherapy plus osimertinib should be used in a select minority of elderly patients, keeping in mind that the identification of elderly patients should focus more on frailty, comorbidities, and functional status rather than on chronological age alone. In other words, neither of the two intensified regimens can be generalized to elderly patients given their extremely limited representation in both trials. Furthermore, the increased time toxicity (greater treatment burden, infusion time, hospital visits, and adverse event management) and financial toxicity associated with the combination regimens are undoubted disadvantages particularly relevant to older adults. On the contrary, it is important to underline that specific scenarios exist and can be recognized in which older adults might still be appropriate candidates for intensified therapy. Examples include biologically younger or functionally robust older adults with preserved organ function, those with minimal comorbidity or polypharmacy and with high-risk clinical or molecular features such as high tumour burden or early CNS involvement, or circumstances in which strong patient preference and adequate support systems allow for safe administration of more intensive regimens. The topic of performance status (PS) 2 patients is even more complex and ambiguous. In both the MARIPOSA and FLAURA 2 trials, only PS 0-1 patients were enrolled. However, in chemo-immunotherapy trials [30], the take-home messages and conclusions from authors for physicians in every-day clinical practice do not specify the PS and concern the whole population, in this case of EGFR-mutant advanced NSCLC patients. Real-world data indicate that advanced EGFR-mutant NSCLC patients have a poorer prognosis than the patients included in the clinical trials. In a real-world cohort of 311 patients, the median PFS was 12.4 months, ECOG PS-2 was reported in 29.4% of patients, and 26.3% of patients had symptomatic brain metastases, whereas these patients were excluded from the FLAURA study [31]. In another retrospective analysis performed in a real-world series of 56 patients with advanced EGFR-mutant NSCLC treated with osimertinib, only 45% of them were defined by the authors to be FLAURA-eligible [32]. In fact, 25% of patients had a PS of 2 or higher (patients in the FLAURA trial only had a PS of 0 or 1) and the median age was more advanced (68 years, range 35–103 years) than in the FLAURA trial, while tumour sites and CNS involvement were similar to in the FLAURA trial. However, efficacy outcomes were also reported to be successful in this real-world series, with a median time to treatment discontinuation of 16.9 months (95% CI: 12.6–35.1). The median TTD results were 31.1 months in the FLAURA-eligible cohort and 12.2 months in the FLAURA-ineligible cohort. The median overall survival for all patients was 32.0 months (95% CI: 15.7–not reached), not reached for the FLAURA-eligible cohort, and 16.5 months for the FLAURA-ineligible cohort. In conclusion, these real-world data show that the efficacy of osimertinib is maintained in real-word contexts, but also confirm that real-world series of NSCLC patients are very different in terms of clinical characteristics from patients enrolled in clinical trials. This difference can result in different efficacy and safety outcomes, mainly for treatments with worse toxicity profiles than osimertinib. Some real-world data have also been reported for the combination of platin-based chemotherapy plus third-generation EGFR-TKIs (osimertinib, aumolertinib, or furmonertinib). In a single-centre retrospective study, data on 382 patients were described [33]. Patients had a PS of 0 or 1, as in the FLAURA 2 trial, and for 29% of them there was CNS involvement at baseline. The efficacy reported in this retrospective analysis was similar to that reported in the FLAURA 2 trial, with a median PFS of 30.9 months (95% CI: 27.5–39.3) and a median OS of 53.9 months (95% CI: 46.2–not reached). Antitumor activity was also relevant, as expected, with an overall response rate of 76.4% and a disease control rate of 98.2%. In terms of toxicity profile, 35.9% of the patients experienced grade 3 or higher adverse events, with myelotoxicity being the most frequent grade 3 adverse event. Thus, efficacy and safety profile in this real-world series were reported to be superimposable to that described in the FLAURA 2 trial. No real-world data have been reported for amivantamab plus lazertinib, but real-world data have been reported for amivantamab as single agent and combined with osimertinib in 61 pretreated advanced EGFR-mutant NSCLC patients (with common, uncommon, and exon 20 insertion mutations) [34]. Antitumor activity was relevant for the pretreated population, with a response rate of 45.2% and disease control rate of 64.3%. More importantly, adverse events reported with amivantamab were similar to those previously described, with no additional toxicities with the combination of osimertinib with amivantamab, which was defined by authors as safe and feasible in this real-world series of patients. In conclusion, if we are obliged to be very precise, we should affirm that the standard of care for PS 2 patients affected by advanced EGFR-mutant NSCLC is yet to be determined, with more real-world data supporting the use of osimertinib alone. We are conscious that in clinical practice, especially for young patients, physicians often extend the application fields of clinical trials as guidelines often omit the PS limitations. However, it should be underlined that clear scientific evidence of the superiority of the combination treatments over osimertinib for PS 2 patients does not exist, and that in PS 2 patients the combinations should be used cautiously and in selected patients only (specifically young patients with few comorbidities).

## 6. When Is Osimertinib Still the Best Option? The Importance of Well-Structured Shared Decision Making in Treatment Choice

In addition to the majority of elderly patients and patients with poor PS, there is another subgroup of patients for whom osimertinib is still the best option: patients that prefer and require a less-toxic oral therapy, even if less efficacious, after well-structured shared decision making. In fact, clinical and molecular prognostic factors, useful for choosing among more toxic and efficacious versus less toxic and efficacious treatments in EGFR-mutant NSCLC, are not enough to determine the best treatment option. Thus, individual patient opinion within shared decision making (SDM) becomes a crucial approach to choose the best treatment for every single patient [35,36]. Structured and validated tools for SDM do not yet exist for this clinical context; thus, in our opinion it is time to improve on this issue [37,38]. Unfortunately, an evidence-based recognized approach for SDM is not yet available. However, some considerations and personal opinions can be drawn. For optimal SDM, before speaking about candidate treatments, in our opinion, the physician should briefly interview the patient on their social, familiar, economic, and working situation. It is very important for the physician to know about the patient’s life to give the best suggestion in the phase of shared decision making. Then, the first element to clarify and explain is the efficacy of the available therapeutic options in terms of difference in median overall survival or survival rate (at 3 or 5 years). If available, the probability of being alive at 5 years is an outcome better understood by patients, and also the preferred outcome to achieve (the probability of being a long survivor). The second element is the description of the safety profile of therapeutic options (particularly symptomatic toxicities and visible adverse events like skin toxicity), route of administration (oral versus subcutaneous versus intravenous), and number of hospital visits per month and per year. In the case of amivantamab plus lazertinib, skin toxicity must be disclosed, as well as the risk of thrombovenous embolism and of infusion-related reactions (specifying their reduced rate with a subcutaneous route of administration, however) and the polypharmacotherapy needed for the prophylaxis of these adverse events. Concerning platin-based chemotherapy plus osimertinib, the rate of myelotoxicity (with the risk of life-threatening toxicities requiring hospitalization) has to be discussed, as well as the prolonged hospital stay for intravenous administration of chemotherapy. After discussing efficacy and toxicity information, the third phase should be a fusion of the two previous phases, with the exploration of a psychological approach towards the extent of the prolongment of survival that the patient would intend to trade-off with the increased risk of toxicities (Table 2). Moreover, concerning well-conducted shared decision making, it is important to underline that regional differences in logistics may exist and influence the type of communication. In some regions, limitations in infusion access, cost structures, available support systems, or patient expectations may result in osimertinib monotherapy being chosen more frequently.

## 7. EGFR-Mutant NSCLC: Beyond First-Line Treatment of Advanced Disease

While searching for and discussing the best treatment options for first-line treatment of advanced disease, in the last 5 years the management of early and locally advanced stage EGFR-mutant NSCLC has radically changed [39]. The third-generation EGFR-TKI osimertinib has been introduced in clinical practice as an adjuvant treatment for three years after surgery, immediately or after adjuvant chemotherapy, improving survival outcomes. The phase III randomized ADAURA trial demonstrated a clinically and statistically significant benefit in disease-free survival, with a hazard ratio of 0.23 (95% CI, 0.18–0.30) in stage II-IIIA disease, an 82% reduction in the risk of central nervous system recurrences or death, and also a significant overall survival benefit with osimertinib therapy versus placebo [40,41]. Moreover, osimertinib has also been demonstrated to improve outcomes of chemotherapy as a neoadjuvant treatment in early stages and to improve survival as a maintenance treatment after chemo-radiotherapy in locally advanced stages. In the neoadjuvant setting, the phase III randomized NeoADAURA trial randomized patients with stage II-IIIB resectable EGFR-mutant NSCLC to receive neoadjuvant osimertinib with or without chemotherapy versus chemotherapy alone prior to surgical resection [42]. Following surgery, systemic therapy-eligible patients (over 90%) were also treated with adjuvant osimertinib (i.e., a perioperative approach). Early results, with a median follow up of approximately 14 months, show that neoadjuvant osimertinib achieves a significant improvement in major pathologic response rates (approximately 25% versus 2%, respectively) compared to chemotherapy alone. For patients with unresectable stage III EGFR-mutant NSCLC, the phase III randomized trial named LAURA has established a new standard of care [43]. After chemo-radiotherapy, patients were randomized to receive osimertinib or placebo, and patients treated with osimertinib achieved a statistically and clinically significant progression-free survival benefit of 39.1 months versus 5.6 months (HR 0.16, 95% CI, 0.10–0.24). Osimertinib as maintenance after chemo-radiotherapy is the new standard of care for locally advanced EGFR-mutant NSCLC. Another topic being developed recently is local consolidative therapy (surgery or radiotherapy) in non-progressor patients treated with osimertinib in the first-line setting in order to delay systemic progression and gain a further benefit in overall survival. The phase II NORTHSTAR trial randomized patients treated with osimertinib to receive or not receive local consolidation therapy when responding to treatment with osimertinib (non-progressive disease), demonstrating a very promising benefit in progression-free survival (25.3 vs. 17.5 months; HR 0.66) favouring the local consolidation therapy, among patients with both oligometastatic and polymetastatic disease [44]. However, only when the survival outcomes of this approach are reported will its role in the first-line treatment of advanced EGFR-mutant NSCLC be clarified. Simultaneously, second- and third-line phase III randomized trials are proposing new treatment options for advanced disease after EGFR-TKI-based treatments, with antibody–drug conjugates being, at the moment, the ones at the more advanced stage of development [39]. In the near future, the first-line treatment scenario will also change for EGFR-mutant diseases progressing during or after adjuvant osimertinib treatment in the early stage or maintenance osimertinib treatment in the locally advanced stage. The choice of first-line option in these cases will be strongly influenced by the progression-free interval time; however, it will favour combination treatments more than in naïve patients. Similarly, the availability of antibody–drug conjugates as valuable second- and third-line options may influence the type of first-line choice in different strategies of treatment sequencing, and also the timing of second-line treatment initiation, especially, in oligoprogressors.

## 8. Conclusions

Patients affected by EGFR-mutant advanced NSCLC now have the possibility to be treated with two new combinations with further improved efficacy outcomes compared with those of osimertinib alone. This chance has to be considered a great opportunity, and, in our opinion, should always be proposed to all PS 0-1 young patients, and to a minority of the remaining part of this population. Osimertinib should be considered the standard treatment for PS > 2 patients, for non-selected elderly patients and for all the patients , and for all the patients that after well-performed shared decision making prefer or require a less efficacious but less toxic treatment. Evidence-based data is the basis and the main part of making a clinical choice between combinations and monotherapy and between the two combinations available, but it is not sufficient at the moment. The remaining part, that sometimes becomes the main concern, must derive from open communication with the patient. Only by integrating data from trials with a well structured shared decision-making process can the best-tailored treatment choice be made for every single patient.

## Figures and Tables

**Table 1:** Main activity, efficacy, and toxicity outcomes of osimertinib in FLAURA trial, amivantamab plus lazertinib in MARIPOSA trial, and osimertinib plus platin-based chemotherapy in FLAURA 2 trial (no formal comparisons possible, only descriptive use).

Regimen (Trial)	RR (%)	Median PFS (Months)	Median OS (Months)	3-Year OS Rate (%)	Rate of AEs ≥ 3 (%)
Osimertinib (FLAURA)	80	18.9	38.6	54	42
Amivantamab plus lazertinib (MARIPOSA)	86	23.7	Not reached	60	80
Osimertinib plus platin-based chemotherapy (FLAURA 2)	83	25.5	47.5	63	64

Note: RR: response rate; PFS: progression-free survival; OS: overall survival; AEs: adverse events.

**Table 2:** Clinical factors that, combined with shared decision making, may influence final treatment decisions in different subgroups of patients with advanced EGFR-mutant NSCLC (personal opinions, to be considered only suggestions useful for discussion and own final personal decision).

	Young and Fit Pts with Favour Progn Factors	Young and Fit Pts with Unfavour Progn Factors	Young and Unfit (for Comorbidities) Pts with Favour Progn Factors	Young and Unfit (for Disease Burden) Pts with Unfavour Progn Factors	Elderly and Fit Pts	Elderly and Unfit Pts (for Comorbidities)	Elderly and Unfit Pts (for Disease Burden)
Osimertinib			First choice		First choice	First choice	First choice
Amivantamab–lazertinib	First choice	First choice		First choice			
Platin-based chemotherapy plus osimertinib	First choice	First choice		First choice			
Osimertinib after SDM	Possible first choice	Second choice, only if clearly preferred by pts		Second choice			
Amivantamab–lazertinib after SDM			Possible first choice		Possible first choice		Second choice
Platin-based chemotherapy plus osimertinib after SDM			Possible first choice		Possible first choice		Second choice

Note. Pts: patients; favour progn factors: favourable prognostic factors; unfavour progn factors: unfavourable prognostic factors; SDM: shared decision making.

## Data Availability

No new data were created or analyzed in this study.
