# Correction: Maione et al. Amivantamab Plus Lazertinib and Platin-Based Chemotherapy Plus Osimertinib in EGFR-Mutant NSCLC: How to Choose Among Them and When Is Monotherapy with Osimertinib Still the Best Option? *Curr. Oncol.* 2026, *33*, 54

**DOI:** 10.3390/curroncol33080440

**Published:** 2026-07-23

**Authors:** Paolo Maione, Francesco Jacopo Romano, Cesare Gridelli

**Affiliations:** 1 Division of Medical Oncology, S.G. Moscati Hospital, 83100 Avellino, Italy; cgridelli@libero.it; 2 Division of Medical Oncology, Cardarelli Hospital, 80131 Naples, Italy; francesco_jacopo@libero.it

## Text Correction

There was an error in the original publication [1]. The number of patients aged more than 75 years in the MARIPOSA trial was wrong.

A correction has been made to Section 5.3. Elderly Patients, Poor PS Patients, and Real-World Data. The CORRECTED paragraph appears below:

Although both the MARIPOSA and FLAURA 2 trials enrolled patients without an upper age limit, the proportion of those aged over 75 was 12% (n = 104/858) in the MARIPOSA trial (the same information for FLAURA 2 trial is not available but we think it should be similar due to the platin-based chemotherapy included in the trial) [8]. Thus, in our opinion, MARIPOSA and FLAURA 2 results should not be acritically generalized to the entire elderly population aged more than 75 years, but they should be applied to patients with clinical characteristics (especially in terms of PS and comorbidities) similar to those of elderly patients enrolled in these two trials [29].

The authors state that the scientific conclusions are unaffected. This correction was approved by the Academic Editor. The original publication has also been updated.

## References

[1] Maione P., Romano F.J., Gridelli C. (2026). Amivantamab Plus Lazertinib and Platin-Based Chemotherapy Plus Osimertinib in EGFR-Mutant NSCLC: How to Choose Among Them and When Is Monotherapy with Osimertinib Still the Best Option?. Curr. Oncol..

